# Supplementary material for: Polycystin-1 Is a Crucial Regulator of BIN1 Expression and T-Tubule Remodeling Associated with the Development of Dilated Cardiomyopathy
Source: Int J Mol Sci. 2022 Dec 30;24(1):667. doi: 10.3390/ijms24010667 (PMC9820588; doi:10.3390/ijms24010667)
Supplement: Supplementary file 1 [file ijms-24-00667-s001.zip › Supplementary Figure S1.pdf]

Supplementary Figure S1

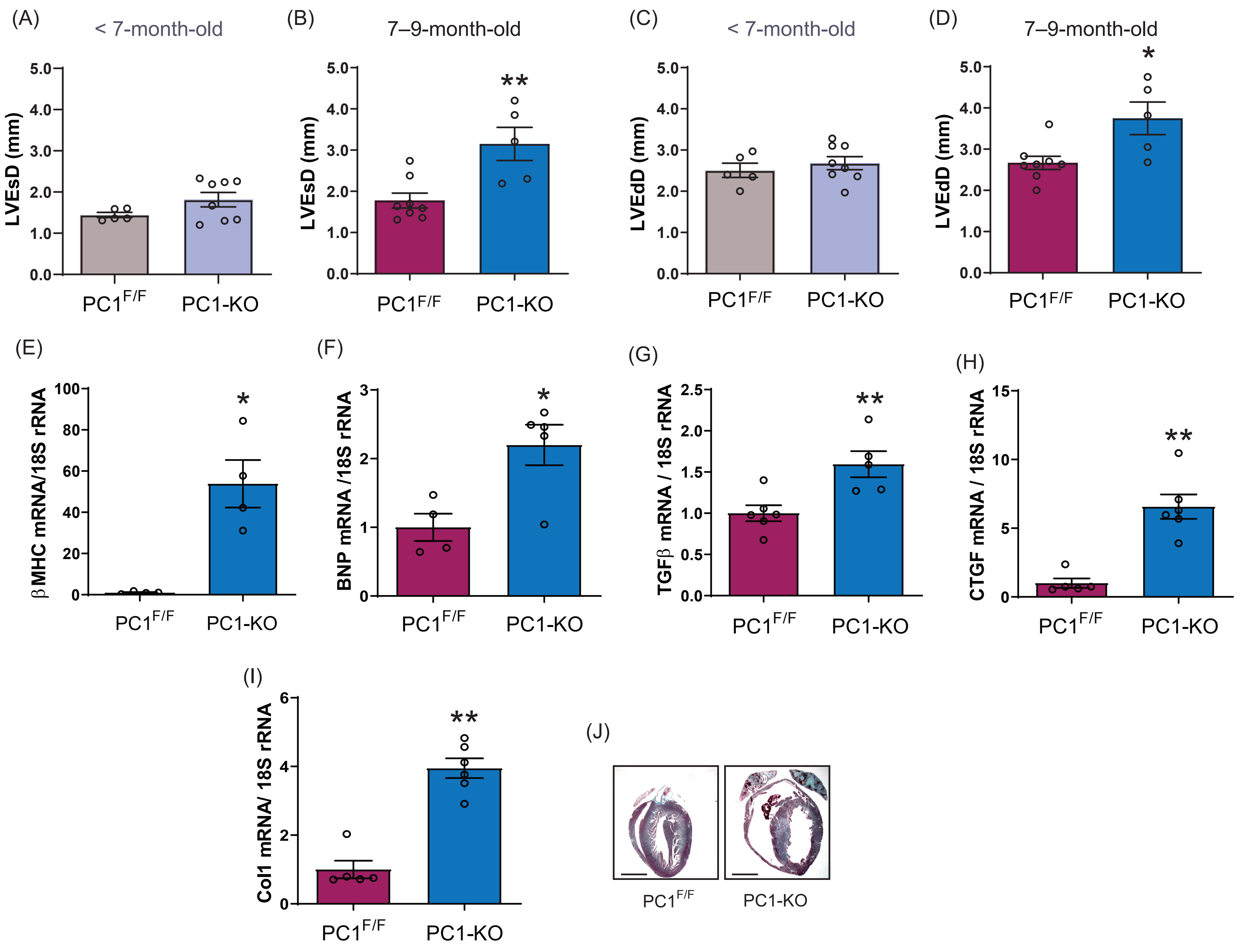

Supplementary Figure S1. (**A**,**B**) Left ventricular end-systolic (LVEsD) and (**C**,**D**) diastolic (LVEdD) diameter are depicted for hearts < 7-months in PC1F/F and PC1-KO mice (n = 5–8). Bar graphs of β-MHC (**E**, n = 4), BNP (**F**, n = 4–5), ANP (**G**, n = 5–6), CTGF (**H**, n = 5–6) and Col1 (**I**, n = 5–6) mRNA for PC1F/F and PC1-KO mice 7–9-months of age. (**J**) Representative Masson's trichrome-stained 4-chamber cross sections of hearts from PC1F/F and PC1-KO mice at 7–9-months of age. Values shown are the means ± SEM and were analyzed using the Student *t* test. * $p < 0.05$; ** $p < 0.005$ vs. PC1F/F.
