# Supplementary material for: Polycystin-1 Is a Crucial Regulator of BIN1 Expression and T-Tubule Remodeling Associated with the Development of Dilated Cardiomyopathy
Source: Int J Mol Sci. 2022 Dec 30;24(1):667. doi: 10.3390/ijms24010667 (PMC9820588; doi:10.3390/ijms24010667)
Supplement: Supplementary file 1 [file ijms-24-00667-s001.zip › Supplementary Figure S2.pdf]

Supplementary Figure S2

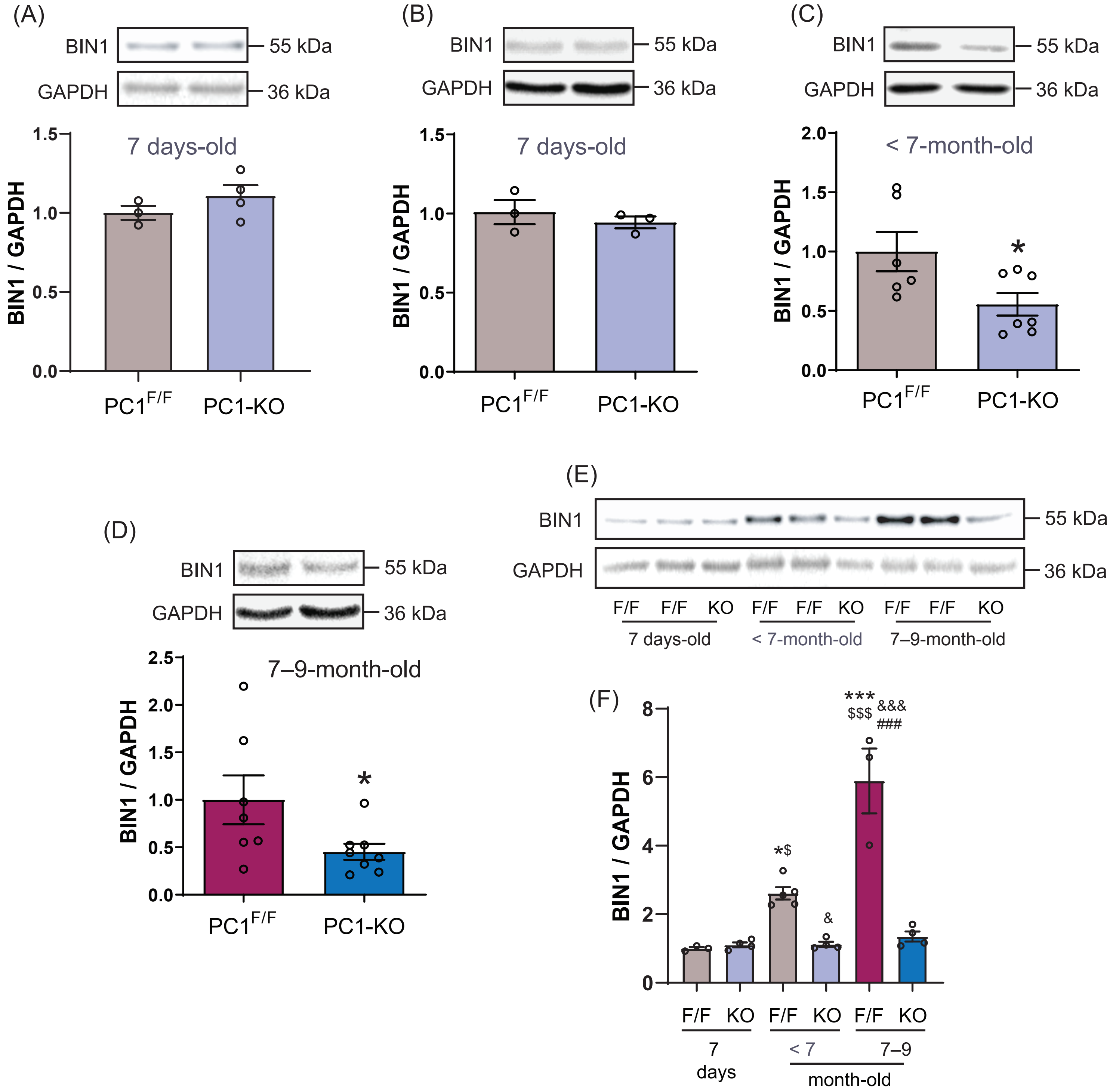

Supplementary Figure S2. Representative western blots and quantification of BIN1 in cardiac tissue from PC1F/F and PC1-KO mice 7-days; (**A**) ab185950 and (**B**) ab95022 (n = 3–4), < 7-months (**C**, n = 6–7) and 7–9-months (**D**, n = 7–8) of age. Values shown are the means ± SEM and were analyzed using the Student *t* test. * $p < 0.05$ vs. PC1F/F. (**E**) Representative western blots of BIN1 and GAPDH. (**F**) Comparison of the BIN1/GAPDH ratio for PC1F/F and PC1-KO (n = 3–5) mice of different ages. Values shown are the means ± SEM and were analyzed by one-way ANOVA followed by a Tukey's test. * $p < 0.05$; *** $p < 0.001$ vs. 7-days-olf PC1F/F, $ $p < 0.05$; $$$ $p < 0.001$ vs. 7-days-olf PC1-KO, & $p < 0.05$; &&& $p < 0.001$ vs. <7-months-olf PC1F/F; ### $p < 0.001$ vs. 7-9-months-old PC1-KO mice.
