# Supplementary material for: Polycystin-1 Is a Crucial Regulator of BIN1 Expression and T-Tubule Remodeling Associated with the Development of Dilated Cardiomyopathy
Source: Int J Mol Sci. 2022 Dec 30;24(1):667. doi: 10.3390/ijms24010667 (PMC9820588; doi:10.3390/ijms24010667)
Supplement: Supplementary file 1 [file ijms-24-00667-s001.zip › Supplementary Figure S3.pdf]

Supplementary Figure S3

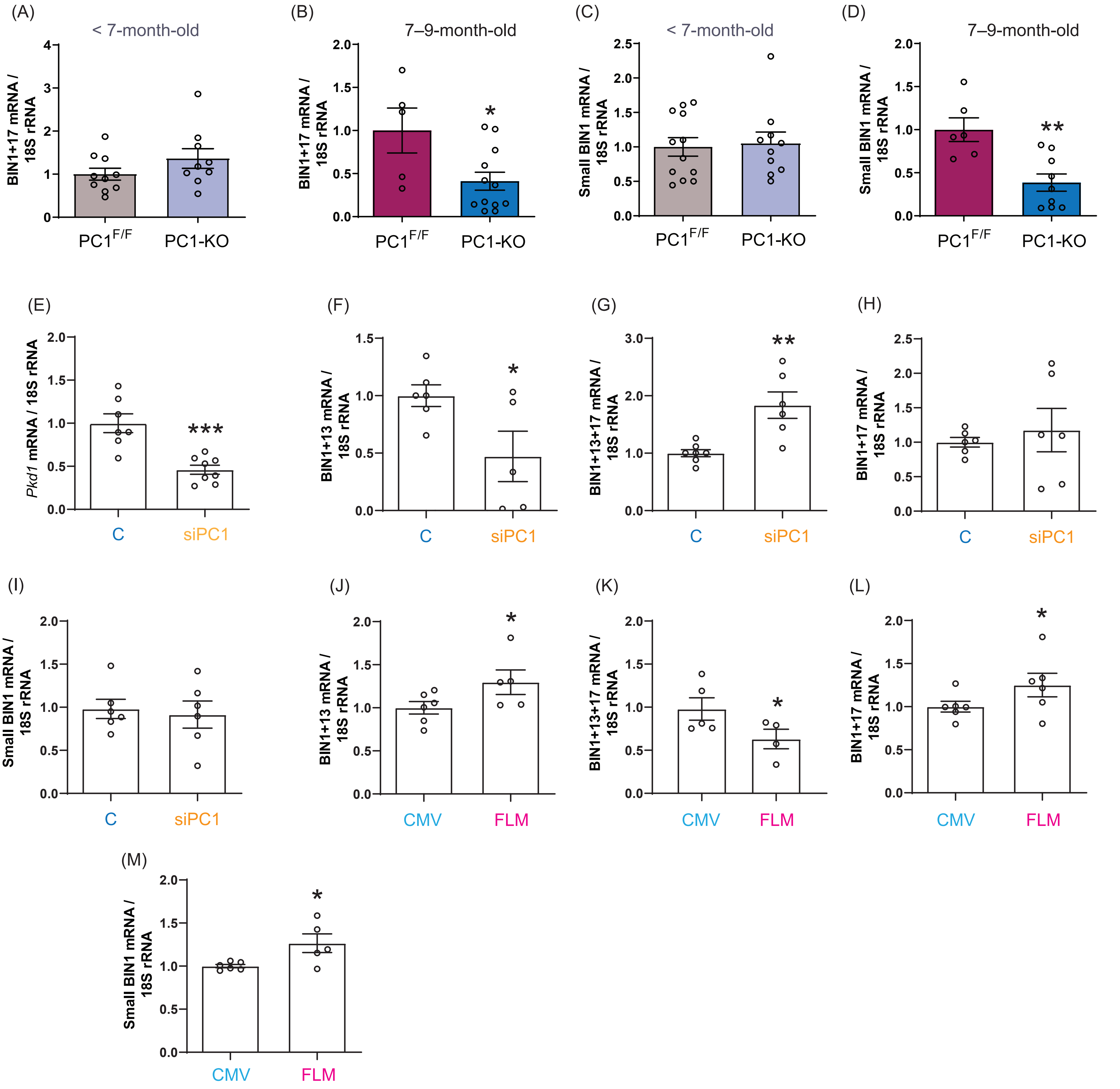


**Supplementary Figure S3.** Quantification of BIN1 isoform mRNA; BIN1+17 at <7-months (**A**, n = 9–10) and 7–9-month (**B**, n = 5–12) of age. Small BIN1 mRNA for mice < 7-months (**C**, n = 10–12) and 7–9-month (**D**, n = 6–9) of age. PC1 mRNA in NRVMs transfected with PC1 specific siRNA (siPC1), (**E**, n = 7–8). BIN1 isoforms mRNA in control (C) and siPC1 NRVMs: BIN1+13 (**F**, n = 5–6), BIN1+13+17 (**G**, n = 6–7), BIN1+17 (**H**, n = 6) and small BIN1 (**I**, n = 6). Bar graph of BIN1+13 (**J**, n = 5–6), BIN1+13+17 (**K**, n = 4–5), BIN1+17 (**L**, n = 6) and small BIN1 (**M**, n = 5–6). Values shown are the means ± SEM and were analyzed using the Student *t* test. * $p < 0.05$; ** $p < 0.005$; *** $p < 0.001$ vs. PC1F/F, control (C) or empty vector cytomegalovirus (CMV).
